# Production of Different Mushroom Protein Hydrolysates as Potential Flavourings in Chicken Soup Using Stem Bromelain Hydrolysis 

**DOI:** 10.17113/ftb.57.04.19.6294

**Published:** 2019-12

**Authors:** San-San Ang, Mohammad Rashedi Ismail–Fitry

**Affiliations:** Department of Food Technology, Faculty of Food Science and Technology, Universiti Putra Malaysia, 43400 UPM Serdang, Selangor, Malaysia

**Keywords:** stem bromelain, mushroom protein hydrolysates, potential flavourings, chicken soup

## Abstract

The pleasant taste of edible mushrooms, which is attributed to their high protein content, makes them an attractive source for the production of protein hydrolysates with good taste properties. In the present work, different mushroom protein hydrolysates were produced from shiitake, oyster, bunashimeji and enoki mushrooms using stem bromelain hydrolysis at 0.5% (*m*/*m*) enzyme/substrate ratio at pH=6.5 and 40 °C for 20 h. The produced liquid mushroom protein hydrolysate yielded 0.77–0.92% crude protein (p>0.05). Bunashimeji mushroom protein hydrolysate was the lightest in colour, while shiitake mushroom protein hydrolysate was the darkest (p<0.05). Enoki mushroom protein hydrolysate had the highest dry matter content. There was no significant difference in the degree of hydrolysis among different mushroom protein hydrolysates (53.52–67.13%, p>0.05), with the highest yield of bunashimeji and the lowest of shiitake mushroom protein hydrolysate (p<0.05). Preference test of chicken soup with added different mushroom protein hydrolysates was performed using 58 untrained panellists to evaluate their taste-enhancing effect, compared to monosodium glutamate (MSG). Soup with MSG had the highest score for the tested attributes, while soups with bunashimeji and oyster mushroom protein hydrolysates showed higher aroma, taste, mouthfeel and overall preference scores than negative control, which contained neither MSG nor any of the hydrolysates (p<0.05). This finding suggests that bunashimeji and oyster mushroom protein hydrolysate have the potential to be used as taste enhancers in food applications.

## INTRODUCTION

Apart from umami amino acids, protein peptides or hydrolysates with taste-enhancing properties have started drawing attention in recent years in the midst of increasing demand for natural taste enhancers in food products. Protein hydrolysates are known as a mixture of oligopeptides, peptides and free amino acids that are released from protein molecules by partial or extensive hydrolysis through chemical cleavage using acid or alkali, proteolytic bacteria or proteolytic enzymes (*1*). At present, more than 50 umami peptides from different sources such as fish, soybean, peanut and beef have been reported and their sequences identified (*2*).

The relatively high protein content in edible mushrooms makes them an attractive source to produce protein hydrolysates for food applications (*3*). The characteristic umami, a broth-like taste of edible mushrooms, was found to be associated with the presence of monosodium glutamate (MSG)-like amino acids - aspartic acid (Asp) and glutamic acid (Glu), as well as 5'-nucleotides (*4*). Palupi *et al.* (*5*) reported the sensory properties of protein hydrolysates of paddy mushrooms (*Volvariella volvaceae*) obtained with commercial protease Protamex^TM^. On the other hand, Lotfy *et al.* (*6*) produced beef-like flavouring from protein hydrolysates of portobello mushrooms (*Agaricus bisporus*) using Flavourzyme and Alcalsase enzymes. Another study reported that 18 and 20 h of papain hydrolysis resulted in hydrolysates from oyster, abalone and shiitake mushrooms with the highest total amino acid content (*7*).

Protein-digesting proteases constitute approx. 60% of the market share in the global enzyme market. Bromelain enzyme from pineapple (*Ananas comosus*) is known as one of the proteases that is gaining increasing industrial interest due to its high versatility and commercial value. The most common food application of bromelain is as meat tenderiser that breaks the cross-links between myofibrillar meat proteins to give meat better eating quality. Besides, bromelain is also used in the baking industry as dough improver, where it degrades the gluten structure to give dough the relaxation effect for better texture in baked goods (*8*).

There are four types of pineapple proteases: stem bromelain (EC 3.4.22.32), fruit bromelain (EC 3.4.22.33), ananain (EC 3.4.22.31) and comosain (*9*). Stem bromelain, optimally working under pH=6.5-8.0 and temperature 40-60 °C (*8*, *9*), was reported to have cleavage preference for glutamic and aspartic acids, which makes it suitable for the production of protein hydrolysates that release umami amino acids and peptides (*10*). In a study by Maehashi *et al.* (*11*), chicken protein hydrolysate with umami taste and high content of free glutamic acid was produced using bromelain hydrolysis. Sonklin *et al.* (*12*), who produced mung bean protein hydrolysate using bromelain enzyme, reported the bouillon, salty, sour and umami taste of the protein hydrolysate, and suggested that the enzyme could be used as the precursor for producing processed or savoury flavours.

In the present work, shiitake (*Lentinus edodes*), oyster (*Pleurotus ostreatus*), bunashimeji (*Hypsizygus tessellatus*) and enoki (*Flammulina velutipes*) mushrooms were selected to produce protein hydrolysates by stem bromelain hydrolysis. Shiitake and oyster mushrooms are among the most frequently cultivated mushroom varieties in Malaysia, while enoki and bunashimeji mushrooms are widely available on the local market (*13*). The aims of the present work are to examine the physicochemical properties of stem bromelain-treated mushroom protein hydrolysates, and to evaluate their taste-enhancing properties by using chicken soup as an application base, in order to demonstrate their potential use as a natural taste enhancer for food applications.

## MATERIALS AND METHODS

### Materials

Fresh shiitake, oyster, bunashimeji and enoki mushrooms purchased from Tesco hypermarket, Putrajaya, Malaysia, were cleaned and air-dried in a laboratory oven (Isotherm^®^ OFA 110-8; Esco, Hatboro, PA, USA) at (50±1) °C for 7-8 h. The moisture content of dried mushrooms was examined using halogen moisture analyser (HR83; Mettler Toledo, Greifensee, Switzerland) (*14*). Dried mushrooms were then cut into small pieces of about 1 cm^2^, packed into aluminium bags and stored at room temperature until further use.

Bromelain (EC 3.4.22.32, ≥3 U/mg) from pineapple stem was purchased from Sigma-Aldrich, Merck, St. Louis, MO, USA. Chemicals and reagents used for analysis were of analytical grade. NaOH, H_3_BO_3_, Na_2_SO_4_ and anhydrous CuSO_4_ were purchased from Merck Millipore, Darmstadt, Germany. HCl 37%, CH_2_O 37-41%, methyl red, methylene blue and phenolphthalein were purchased from Fisher Scientific, Hampton, VA, USA. H_2_SO_4_ 95-97% and approx. 95% C_2_H_5_OH were purchased from QRec, Rawang, Malaysia, while SeO_2_ was purchased from Scharlau, Barcelona, Spain. Fat-free chicken broth powder (NCB400; Proliver, Olen, Belgium), which does not contain additives, and other soup ingredients (salt, corn starch, herbs and spices) were used in the preparation of chicken soup.

### Crude protein content and pH measurement

The crude protein content in dried mushrooms was determined by the Kjeldahl method using the conversion factor of 4.38 (*3*, *15*). The dried mushrooms were mixed with distilled water at a ratio 1:15 (*m*/*V*). The mixture was homogenised at 11 000 rpm for 1 min (Ultra-Turrax^®^, T25 basic; IKA, Staufen, Germany) to produce mushroom slurry. The pH of the mushroom slurry was measured using a pH meter (PC700; Eutech Instruments, Singapore) (*7*).

### Production of mushroom protein hydrolysates

The mushroom protein hydrolysates were produced following the methods of Sukkhown *et al.* (*16*) and Wang *et al.* (*17*) with some modifications. The produced mushroom slurries were adjusted to pH=8.0 using 2 M NaOH, and stirred at 350 rpm for 1 h on a magnetic stirring plate (Fisher Isotemp^®^; Fisher Scientific) (*18*). Next, the slurry was adjusted to optimal enzymatic pH=6.5 using 2 M HCl. Bromelain enzyme was applied at 0.50% of dried mushroom mass, in which the enzyme was dispersed in distilled water at a ratio of 1:5 (*m*/*V*) prior to addition.

The mushroom slurry was incubated for 20 h at 40 °C in a water bath shaker (model 903; Hotech Instruments, New Taipei, Taiwan) at 100 rpm, followed by enzyme inactivation in hot water bath at 85 °C for 20 min. The hydrolysed slurry was cooled to room temperature, adjusted to pH=7.0 by using 2 M NaOH and centrifuged (Sorvall^®^ Primo R; Thermo Scientific, Waltham, MA, USA) at 4000 rpm and 25 °C for 30 min. The supernatant was collected as mushroom protein hydrolysate, adjusted to pH=7.0 using 2 M NaOH and stored at -20 °C until further analyses. Protein hydrolysate of each mushroom type was produced in triplicate (*17*, *19*).

### Physicochemical properties of mushroom protein hydrolysate

The colour of the hydrolysate was measured using a colourimeter (ColorQuest XE; HunterLab, Reston, VA, USA), illuminant D_65_, 10° viewing angle in 20-mm glass transmission cell, and expressed as CIE L*, a* and b* (*12*). The pH of the hydrolysate at the end of bromelain hydrolysis was measured using a pH meter (PC700; Eutech Instruments) (*7*). The dry matter content of the hydrolysate was determined using the HR83 (Mettler Toledo) halogen moisture analyser (*20*). Its total nitrogen and crude protein content were examined using the Kjeldahl method (*8*, *15*).

### Yield of mushroom protein hydrolysate

The yield of the hydrolysate was expressed as the percentage of its dry matter content in relation to the mass of dried mushroom used for the bromelain hydrolysis (*21*, *22*):

*Y*=(*m*_1_/*m*_2_)·100 /1/

where *m*_1_ is the dry matter mass in mushroom protein hydrolysate (g) and *m*_2_ is the mass of dried mushroom used (g).

### Degree of hydrolysis of mushroom protein hydrolysate

The degree of hydrolysis (DH) of the hydrolysate was determined by formol titration according to the following equations:

*w*(free amino group)=[(*c*·*V*·14.007)/(*m*_3_·1000)]·100 /2/

and

DH=[(*w*(free amino group)/*w*(total nitrogen)]·100 /3/

where *c* is the molar concentration of NaOH, *V* is the volume of 0.1 M NaOH (mL), *m*_3_ is the mass of mushroom protein hydrolysate (g), and 14.007 is the molar mass of nitrogen. In this procedure, 5.0 g of the hydrolysate were mixed with distilled water and adjusted to pH=7.0 with 1 M NaOH. Then, 10 mL of 38% (*V*/*V*) CH_2_O solution (pH=8.5) were added and the mixture was left for 5 min for the formaldehyde reaction. Next, the mixture was titrated with 0.1 M NaOH to the end point of pH=8.5 by using 1% phenolphthalein as the colour indicator (*22*).

### Preparation of chicken soup

All mushroom protein hydrolysates were first adjusted to 3.0% (*m*/*m*) based on respective dry matter content for standardisation of mass fraction, while 0.9 g MSG powder (Ajinomoto, Kuala Lumpur, Malaysia) was dissolved in 29.1 g distilled water to make 3.0% (*m*/*m*) MSG solution. Six samples of chicken soup were prepared: negative control without any taste enhancers, positive control with MSG solution, and four samples with each type of mushroom protein hydrolysate. The chicken soup was made up of (in %): MSG solution or mushroom protein hydrolysate 3.3, chicken broth powder 1.20, salt 0.40, corn starch 0.40, onion powder 0.10, garlic powder 0.07, dehydrated chives 0.05, black pepper powder 0.03 and water 94.42.

### Preference test of chicken soup

A group of 58 untrained sensory panellists was recruited for the sensory preference test. Each panellist was given six cups of 20 mL warm chicken soup labelled with randomised three-digit codes, along with a cup of plain water to cleanse the palate between samples. The chicken soups were analysed using the 9-point hedonic scale for the attributes of aroma, taste, colour, mouthfeel and overall preference of the samples (*23*).

### Physicochemical properties of chicken soups

The colour of chicken soup was measured in glass transmission cell with 20 mm path length using a spectrophotometer (ColorQuest XE; HunterLab), illuminant D_65_, 10° viewing angle, and the measurement was expressed as CIE L*, a* and b*. The pH of the chicken soup was measured using a pH meter (PC700; Eutech Instruments). The measurement of the apparent viscosity of chicken soup was carried out on controlled stress rheometer (RheoStress 600; Thermo Haake, Karlsruhe, Germany) using parallel plate geometry (35 mm diameter) at gap size of 0.5 mm. The shear rate ranging from 1 to 100 s^-1^ was applied for 60 s at a constant temperature of 25 °C. The apparent viscosity of chicken soup at a shear rate of 100 s^-1^ was reported (*24*).

### Statistical analysis

All the analyses and measurements were carried out in triplicate for each type of mushroom protein hydrolysate, and the results were reported as mean value±standard deviation. The obtained data were statistically analysed using a one-way analysis of variance (ANOVA) with a significance level of p<0.05. The statistical software used was Minitab 16 (*25*).

## RESULTS AND DISCUSSION

### Properties of dried mushrooms

Mushrooms are highly perishable due to the high water content of about 90%; therefore, fresh mushrooms were first air-dried to prevent deteriorative microbial growth and enzymatic activity for longer shelf life (*3*, *26*). The drying temperature of 50-60 °C was reported ideal to retain the physicochemical properties of mushrooms (*26*). The moisture content of 10.5-12.0% (Table 1) in dried mushroom samples shows that drying was properly applied for preservation in the present work (*27*).

**Table 1:** Moisture and crude protein content of dried mushroom, and pH of mushroom slurry

Dried mushroom	*w*(moisture)/%	*w*(crude protein)/%	pH of mushroom slurry
Shiitake	(12.0±0.2)^a^	(16.0±2.0)^a^	(6.2±0.2)^b^
Oyster	(11.7±1.1)^a^	(18.2±2.4)^a^	(6.4±0.2)^b^
Bunashimeji	(10.7±0.7)^a^	(14.2±2.9)^a^	(6.55±0.08)^ab^
Enoki	(10.5±1.6)^a^	(15.8±0.8)^a^	(6.88±0.09)^a^

Values are expressed as mean±S.D., *N*=3. Mean values in the same column with different letters in superscript are significantly different (p<0.05) using ANOVA

A lower conversion factor of 4.38 was used for the determination of crude protein content that excluded the non-protein nitrogen present in the mushroom cell wall (*3*). Oyster mushroom showed the highest crude protein content, while bunashimeji mushroom had the lowest, on a dry mass basis. However, the difference was not significant among mushroom types (p>0.05). The pH values of mushroom slurries were within the near-neutral pH range of 6.0-6.7, reported previously for raw mushrooms (*28*).

### Colour and pH of mushroom protein hydrolysates

The result of colour measurement (Table 2) shows that bunashimeji protein hydrolysate was the lightest (highest L*) and the most yellowish (highest +b*), while that of shiitake was the darkest (lowest L*) and the most reddish (highest +a*). In contrast, enoki protein hydrolysate was the least reddish (lowest +a*) and least yellowish (lowest +b*) with p<0.05. However, Banjongsinsiri *et al.* (*7*) produced shiitake and oyster mushroom protein hydrolysates with higher L* and lower a* using papain hydrolysis. In the present work, the enoki protein hydrolysate was also found darker than the fresh mushroom, which was elsewhere reported with L* approx. 85 (*29*). The darker colour of the hydrolysates observed in the present work than in previous studies could be because of the higher extent of non-enzymatic Maillard browning occurring during mushroom drying, which means that they had a higher level of the brown-black pigment called melanin (*30*). In fact, for food applications colour attributes of hydrolysates are desirable to be closest to those of fresh mushrooms. Darker colour hydrolysates could affect consumer acceptance of the food products in which they are applied (*31*).

**Table 2:** Physicochemical properties of bromelain-hydrolysed mushroom protein hydrolysates

Protein hydrolysate	Colour			pH
L*	a*	b*
Shiitake	(30.0±2.4)^c^	(28.1±0.4)^a^	(49.6±3.6)^bc^	(5.6±1.1)^a^
Oyster	(50.7±1.6)^b^	(14.4±1.1)^b^	(59.3±2.9)^ab^	(4.19±0.06)^a^
Bunashimeji	(64.3±5.4)^a^	(11.2±1.6)^c^	(64.4±6.6)^a^	(4.34±0.07)^a^
Enoki	(54.5±5.9)^ab^	(8.0±1.4)^d^	(43.9±2.4)^c^	(4.6±0.3)^a^

Values are expressed as mean±S.D., *N*=3. Mean values in the same column with different letters in superscript are significantly different (p<0.05) using ANOVA

A decrease in the pH from initial optimal enzymatic pH of 6.50 was observed in all hydrolysates at the end of bromelain hydrolysis (Table 2), which could be due to the dissociation of H^+^ ions from free amino groups into neutral or basic hydrolysis medium when the peptide bonds were enzymatically cleaved (*32*). The finding is in good agreement with the observation of Banjongsinsiri *et al.* (*7*). In addition, the pH values were not significantly different among the hydrolysates (p>0.05).

### Dry matter content of mushroom protein hydrolysates

In general, mushrooms have low dry matter content due to their high water content (*3*). The dry matter content of the hydrolysates was in the range of 3.24-3.8% (Table 3). Enoki protein hydrolysate had the significantly highest dry matter content, while that of oyster mushroom had the lowest (p<0.05). Fresh enoki was reported with higher dry matter content than fresh shiitake and oyster mushrooms in previous studies (*33*, *34*). This could explain the higher dry matter content of the enoki protein hydrolysate observed in the present work than of shiitake and oyster mushroom protein hydrolysate. In comparison to the dry matter content of fresh shiitake reported by Mattila *et al.* (*34*), the low dry matter content of shiitake protein hydrolysate could be due to the variation in cultivation factors such as temperature and relative humidity of growth environment, the level of watering, and growth substrate used for different sources of mushroom (*34*).

**Table 3:** The yield, dry matter and crude protein content, and degree of hydrolysis (DH) of mushroom protein hydrolysates after bromelain hydrolysis for 20 h

Protein hydrolysate	*Y*/%	*w*(dry matter)/%	*w*_wet_(crude protein)/%	*w*_wet_(total nitrogen)/%	*w*_wet_(amino nitrogen)/ %	DH/%
Shiitake	(35.2±2.2)^b^	(3.4±0.2)^b^	(0.91±0.09)^a^	(0.21±0.02)^a^	(0.11±0.02)^a^	(53.5±3.8)^a^
Oyster	(36.9±0.6)^ab^	(3.24±0.08)^b^	(0.8±0.7)^a^	(0.19±0.02)^a^	(0.11±0.01)^a^	(62.4±2.3)^a^
Bunashimeji	(39.3±3.2)^ab^	(3.47±0.08)^ab^	(0.79±0.07)^a^	(0.18±0.02)^ab^	(0.12±0.01)^a^	(67.1±10.4)^a^
Enoki	(42.5±3.5)^a^	(3.8±0.3)^a^	(0.8±0.3)^a^	(0.15±0.01)^b^	(0.09±0.02)^a^	(65.7±12.7)^a^

Values are expressed as mean±S.D., *N*=3. Mean values in the same column with different letters in superscript are significantly different (p<0.05) using ANOVA

### The crude protein content of mushroom protein hydrolysates

There was no significant difference in crude protein contents among different hydrolysates (p>0.05) (Table 3). The protein contents of shiitake and oyster mushroom were higher than the reported values by Banjongsinsiri *et al.* (*7*) of papain-hydrolysed shitake (0.72%) and oyster mushroom protein hydrolysate (0.60%). In addition, the bromelain hydrolysis time (20 h) in the present work was shorter than their papain hydrolysis time (24 h). The result is consistent with the finding of Guo *et al.* (*35*), who demonstrated that bromelain hydrolysis produced rice protein hydrolysate with higher protein content than papain hydrolysis under respective optimal enzymatic conditions. This implies that bromelain could be a promising enzyme for the commercial production of valuable protein hydrolysates with high protein content.

### Yield of mushroom protein hydrolysates

Enoki protein hydrolysate had the highest yield (42.5%), followed by bunashimeji (39.3%), oyster mushroom (36.9%) and shiitake (35.2%) protein hydrolysate (p<0.05) (Table 3). The yield of protein hydrolysates represents the efficiency of enzymatic hydrolysis in recovering the peptides or free amino acids from raw materials after the process, and the factors affecting yield include types and mass fraction of enzyme used, duration of hydrolysis, pH, and temperature of the hydrolysis medium (*36*). Higher yield is always desirable in terms of economic feasibility for commercial production of protein hydrolysates to be used as ingredients for industrial applications (*37*).

The yield of the hydrolysates was in accordance with their dry matter content, whereby enoki protein hydrolysate with the highest dry matter content gave the highest yield, while that of shiitake with the lowest dry matter content had the lowest yield. This suggests that the bromelain hydrolysis under the set parameters (pH=6.5, 40 °C) resulted in yield that fitted well with the respective dry matter content of the used mushrooms (*21*). Presently, there are no reported data available on the yield of mushroom protein hydrolysate using bromelain hydrolysis. Sonklin *et al.* (*12*) reported approx. 45% yield of mung bean protein hydrolysates after 24 h of bromelain hydrolysis with 18% enzyme mass fraction, which is in agreement with the observation of the present work. On the other hand, Guo *et al.* (*35*), who conducted response surface investigation on bromelain-hydrolysed rice protein, reported 23.66–31.26% yield after 4 h of hydrolysis, which implies that the yield of the hydrolysate was proportional to the time of bromelain hydrolysis.

### Degree of hydrolysis

The degree of hydrolysis (DH) is the percentage of cleaved peptide bonds in relation to the total number of peptide bonds present in the protein. DH has been an important parameter in defining the functional properties of protein hydrolysates in the industry, as well as a useful monitoring tool in controlling the hydrolytic reaction in order to produce hydrolysates with tailored characteristics for end users (*38*).

Bunashimeji protein hydrolysate was shown to have the highest DH (67.1%), followed by enoki (65.7%), oyster mushroom (62.4%) and shiitake (53.5%) protein hydrolysate (Table 3). The difference in DH was not significant among the hydrolysates (p>0.05) due to the constant hydrolytic factors, *i.e*. time, pH, temperature and bromelain mass fraction across all the hydrolysates. A significant difference in bromelain DH was demonstrated by Sonklin *et al.* (*12*), who applied various hydrolysis times and enzyme mass fractions. Therefore, it could be inferred that the bromelain hydrolysis of all the samples in the present work was under control using constant hydrolytic factors.

The observed range of DH was close to the 60% DH recorded by Sonklin *et al*. (*12*) for mung bean protein after 20 h of hydrolysis at 18% enzyme mass fraction. Palupi *et al.* (*5*) observed a lower DH of 19.06–24.59% in paddy mushroom protein hydrolysate. Banjongsinsiri *et al.* (*7*) also reported lower DH values (less than 50%) in papain-hydrolysed shiitake and oyster mushroom hydrolysates after 24 h of hydrolysis. This implies that bromelain is a potential enzyme for the production of mushroom protein hydrolysates with higher DH, which could benefit the tailoring of functional protein hydrolysates.

### Sensory evaluation of chicken soup

Table 4 shows the mean preference scores of 58 untrained panellists for aroma, taste, colour, mouthfeel and overall preference of the chicken soup samples. Soup with MSG had the highest aroma score, while shiitake protein hydrolysate had the lowest. The difference in aroma scores was significant among the chicken soups (p<0.05). The highest aroma score of the soup with MSG is in agreement with the finding of Nishimura *et al.* (*39*), who reported that the addition of umami substances enhanced the aroma sensation of chicken soup. The lowest aroma score of shiitake protein hydrolysate indicated that its characteristic aroma was less preferred in the soup, which could be attributed to the major volatile alcohol compound, 1-octen-3-ol, found in shiitake mushroom that gives strong earthy and herbaceous odour (*40*).

**Table 4:** Preference scores for aroma, taste, colour, mouthfeel and overall preference attributes of chicken soup with the addition of MSG or different mushroom protein hydrolysates

Sample	Colour	Aroma	Taste	Mouthfeel	Overall preference
Control	(5.1±1.7)^a^	(4.5±1.6)^ab^	(4.4±1.6)^b^	(4.7±1.7)^ab^	(4.6±1.5)^ab^
MSG	(5.3±1.7)^a^	(5.0±1.7)^a^	(5.5±1.8)^a^	(5.5±1.8)^a^	(5.6±1.6)^a^
Shiitake	(5.1±1.8)^a^	(3.9±1.8)^b^	(4.3±1.9)^b^	(4.4±2.0)^b^	(4.5±2.0)^b^
Oyster	(5.4±1.9)^a^	(4.6±1.7)^ab^	(4.5±1.9)^b^	(4.7±1.9)^ab^	(4.8±1.9)^ab^
Bunashimeji	(5.4±1.9)^a^	(4.7±2.0)^ab^	(4.7±1.9)^ab^	(4.8±1.9)^ab^	(4.9±1.9)^ab^
Enoki	(5.0±1.8)^a^	(4.4±1.8)^ab^	(4.1±1.6)^b^	(4.6±1.8)^ab^	(4.6±1.7)^b^

Values are expressed as mean±S.D., *N*=58. Mean values in the same column with different letters in superscript are significantly different (p<0.05) using ANOVA. Preference score: 1=dislike extremely, 5=neither like nor dislike, 9=like extremely. Control=chicken soup without taste enhancer, MSG=monosodium glutamate

For taste attribute, the highest score was observed in the soup with MSG and the lowest with enoki protein hydrolysate (p<0.05). MSG is known to enhance the palatability of savoury foods such as meat and fish dishes instead of milk and confectioneries, hence the highest taste score of chicken soup with MSG solution might be due to the umami effect elicited by the l-glutamic acid in the MSG (*41*). The taste scores almost paralleled those of aroma. The correlation between the preference trend of taste and aroma could possibly be due to the integration of taste and olfactory stimuli in the flavour perception of chicken soup by the panellists during the preference test (*42*).

The second-highest taste and aroma score of chicken soup with bunashimeji followed by the soup with oyster mushroom protein hydrolysates compared to negative control shows the taste-enhancing effect of both hydrolysates. This can be explained by the higher DH of the bunashimeji protein hydrolysate that resulted in a higher content of short peptides and free amino acids liberated from the mushroom protein (*43*). There was a positive correlation between the DH and umami taste in minced beef and porcine plasma protein hydrolysate reported by Fu *et al.* (*21*) in their study involving ten different proteases including bromelain.

According to the content of MSG-like glutamic and aspartic acids in cultivated mushrooms reported by Phat *et al.* (*44*), enoki had 5.83 and 1.60 mg/g, shiitake had 9.54 and 1.93 mg/g, while oyster mushroom had 20.0 and 7.66 mg/g glutamic and aspartic acid, respectively. Although enoki protein hydrolysate was observed with higher DH than oyster mushroom and shiitake protein hydrolysates, the lower content of glutamic and aspartic acids in enoki based on the literature could explain the lowest taste score of the chicken soup with its protein hydrolysate. Apart from this, oyster mushroom with a higher content of MSG-like amino acids than shiitake could be the reason why chicken soup with oyster mushroom protein hydrolysate had a higher taste score than that of shiitake. This observation could also be attributed to the higher DH of the oyster mushroom than shiitake protein hydrolysate, which is corroborated by the finding of Banjongsinsiri *et al.* (*7*), who recorded an increase in the content of free glutamic acid and aspartic acid in shiitake and oyster mushroom protein hydrolysates, followed by the increase in the DH using papain hydrolysis. Therefore, it can be inferred that both factors of DH and content of glutamic and aspartic acids synergistically contributed to the taste score of chicken soup assessed in the present work.

However, the taste scores of the chicken soup with bunashimeji and oyster mushroom protein hydrolysate were not significantly higher than the negative control (p>0.05). This could be due to the very low mass fraction of short peptides and free amino acids present in the mushroom protein hydrolysates (3.33%) added to the soup, which was equivalent to only 0.1% (*m*/*m*) of dry solid content made up of mushroom protein, carbohydrate, fibre and ash (*3*). In comparison to the chicken soup with 3.33% (*m*/*m*) MSG, which was equivalent to 0.1% (*m*/*m*) pure l-glutamic acid, the taste-enhancing effect of bunashimeji protein hydrolysate may not be pronounced enough to give a statistical difference in the taste score. Therefore, this suggests that the taste-enhancing property of the mushroom protein hydrolysate should be further consolidated by the increased dry solid content of the hydrolysate in the food application.

There was no significant difference in the colour preference scores (p>0.05). However, it was observed that L*, a* and b* of chicken soups were significantly different (p<0.05) from each other when different mushroom protein hydrolysates were added (Table 5). One possible reason is that the objective colour differences among the chicken soups could be visually undetectable by the panellists due to the lower colour discrimination sensitivity of the naked eye than of the spectrophotometer (*45*).

**Table 5:** The physicochemical properties of chicken soup with the addition of MSG or different mushroom protein hydrolysates

Sample	Colour			pH	*η*/(Pa·s)
L*	a*	b*
Control	(33.4±1.1)^ab^	(5.9±0.4)^bc^	(30.0±0.2)^b^	(6.17±0.01)^b^	(8.4±0.5)^a^
MSG	(34.9±1.4)^a^	(5.6±0.4)^c^	(30.3±0.2)^ab^	(6.20±0.02)^ab^	(8.6±0.2)^a^
Shiitake	(30.0±1.2)^c^	(7.2±0.1)^a^	(31.2±0.3)^a^	(6.23±0.02)^a^	(8.5±0.2)^a^
Oyster	(30.0±0.4)^c^	(6.8±0.3)^a^	(30.7±0.1)^ab^	(6.16±0.04)^b^	(7.8±0.3)^a^
Bunashimeji	(31.7±1.5)^bc^	(6.6±0.4)^ab^	(31.0±0.5)^a^	(6.22±0.02)^ab^	(8.7±0.5)^a^
Enoki	(31.0±0.6)^bc^	(6.8±0.1)^a^	(30.8±0.3)^ab^	(6.20±0.02)^ab^	(8.2±0.2)^a^

Values represent mean±S.D., *N*=3. Mean valuess in the same column with different letters in superscript are significantly different (p<0.05) using ANOVA. Control=chicken soup without taste enhancer, MSG=monosodium glutamate, *η*=apparent viscosity at shear rate 100 s^-1^

On the other hand, the lower L* and higher a* values of the chicken soup with shiitake and oyster mushroom protein hydrolysates were attributed to the darker colour of the two hydrolysates. Meanwhile, the control sample and chicken soup with MSG, which did not contain the hydrolysates, had higher L* (lighter) and lower a* values (less reddish). This indicates that the addition of 3.3% (*m*/*m*) mushroom protein hydrolysate affected the colour of chicken soup, but the colour effect did not influence the colour preference of the soup by sensory panellists.

No significant difference was observed in the viscosity (Table 5) among the chicken soups (p>0.05). However, there was a significant difference observed in the mouthfeel preference scores (p<0.05); chicken soup with MSG had the highest, while soup with enoki protein hydrolysate had the lowest score (Table 4). Besides umami taste, MSG is also known to enhance other food sensory attributes such as thickness, long-lastingness, mouthfulness, impact and mildness (*46*). This could explain the highest mouthfeel score of chicken soup with MSG, and the lowest score with enoki protein hydrolysate, which demonstrated the least taste-enhancing effect, despite the non-significant difference in the objective viscosity measurement. Another similar finding was also reported by Leong *et al.* (*47*), who observed a significant increase in mouthfeel intensity in chicken rice with reduced salt when the umami flavour enhancer was used.

Chicken soup with MSG had significantly the highest overall score (5.6), followed by bunashimeji protein hydrolysate (4.9), oyster mushroom protein hydrolysate (4.8), control (4.6), enoki protein hydrolysate (4.6) and shiitake protein hydrolysate (4.5) (p<0.05). This observation shows that the main drivers in the overall preference level of the chicken soup for the 58 untrained panellists recruited in the sensory evaluation were the attributes of aroma, taste and mouthfeel.

## CONCLUSIONS

With the increasing consumer demand for food products without artificial ingredients or additives, food manufacturers are shifting towards natural ingredients in their product recipes in order to gain competitive market advantage and protect their brand image. Therefore, in view of the negative public perception of the common taste enhancer monosodium glutamate, natural taste enhancers that are easily available and affordable are greatly favoured by the food industries. The present work demonstrated that enzymatic protein hydrolysis using stem bromelain enzyme could give a reasonable yield of mushroom protein hydrolysates, and the high degree of hydrolysis shown by the enzyme is a useful finding for the design of protein hydrolysates with specific properties. Chicken soup with bunashimeji and oyster mushroom protein hydrolysate, which had higher preference scores than negative control, indicated the taste-enhancing property of these hydrolysates. This finding suggests that bunashimeji and oyster mushroom protein hydrolysate could be potential natural taste enhancers for food applications. However, it is suggested to further analyse the amino acid profiles of the hydrolysates by high-performance liquid chromatography to examine the respective content of glutamic and aspartic acids for more objective comparison of their taste-enhancing property. Besides, the molecular mass distribution of short peptides obtained in the stem bromelain hydrolysis can also be determined chromatographically, especially the content of low-molecular-mass peptides. Apart from this, trained panellists who are familiar with the umami taste can be used for the sensory evaluation of end products containing the mushroom protein hydrolysates, in addition to the use of taste profiling instead of preference tests. Furthermore, the liquid hydrolysates can be spray-dried or freeze-dried to produce powdered form for better shelf-life stability, as well as with higher solid content for the ease of application in food products.
